# Low-Cost Optimized U-Net Model with GMM Automatic Labeling Used in Forest Semantic Segmentation

**DOI:** 10.3390/s23218991

**Published:** 2023-11-05

**Authors:** Alexandru-Toma Andrei, Ovidiu Grigore

**Affiliations:** Electronics, Telecommunications & Information Technology Faculty, Polytechnic University of Bucharest, 060042 Bucharest, Romania; ovidiu.grigore@upb.ro

**Keywords:** Convolutional Neuronal Network, semantic segmentation, Gaussian Mixture Model, computer vision, aerial imagery, U-Net, clustering

## Abstract

Currently, Convolutional Neural Networks (CNN) are widely used for processing and analyzing image or video data, and an essential part of state-of-the-art studies rely on training different CNN architectures. They have broad applications, such as image classification, semantic segmentation, or face recognition. Regardless of the application, one of the important factors influencing network performance is the use of a reliable, well-labeled dataset in the training stage. Most of the time, especially if we talk about semantic classification, labeling is time and resource-consuming and must be done manually by a human operator. This article proposes an automatic label generation method based on the Gaussian mixture model (GMM) unsupervised clustering technique. The other main contribution of this paper is the optimization of the hyperparameters of the traditional U-Net model to achieve a balance between high performance and the least complex structure for implementing a low-cost system. The results showed that the proposed method decreased the resources needed, computation time, and model complexity while maintaining accuracy. Our methods have been tested in a deforestation monitoring application by successfully identifying forests in aerial imagery.

## 1. Introduction

Until the 21st century, there was a high demand for imagery data, including satellite, aerial, medical, and so on. However, due to the constant evolution of technology, better sensors and survey platforms have emerged, resulting in a constantly growing pool of data, some of it even available free of charge. The main challenge is how to evaluate this data. Image analysts must have years of training to be both fast and accurate, and even then they specialize in one domain. For example, a military intelligence analyst cannot interpret medical images with success. In recent years, the need for image data interpretation grew, and along with it, machine learning techniques such as Convolutional Neural Networks (CNN). The first notion of CNN was introduced in the late 1980s by Yann LeCun and his colleagues [1], but the groundbreaking point was in 2011 when AlexNet [2] was presented and gave rise to deep CNNs. Besides the eight layers, AlexNet used techniques that might look ordinary currently, such as the ReLU activation function and dropout regularization which had a major impact on the overall accuracy of the network.

After this, infinite possibilities emerged regarding the architecture of a CNN, but a few of them stood out. In 2014, the University of Oxford proposed the Visual Geometry Group (VGG) [3]. Even though it was a very simple architecture comprised of 3 × 3 convolutional filters, it could achieve a much greater depth of up to 19 layers. This way, it could learn more complex information. Next, ResNet [4] introduced the concept of skip connections by creating shortcuts in the architecture. This way it could be trained networks with dozens of layers. Another important feat was the bottleneck architecture, which implemented 1 × 1 convolutional layers before and after bigger filter layers. This method reduced the computation cost and guaranteed the training of deeper CNNs without significant issues.

In 2015, Olaf Ronneberger, Philipp Fischer, and Thomas Brox presented the U-Net architecture [5], an innovative and symmetrical CNN oriented for pixel classification. The scope of the study was biomedical image segmentation but, since then, U-Net has been adapted for use in a multitude of semantic segmentation tasks such as handwriting, medical observations, industry automation, and satellite image segmentation, a domain where the overall study purpose, deforestation monitoring, falls within. Another reason for choosing U-Net over other architectures is its ability to work with fewer images and poor annotation. As will be seen in the next chapter, the dataset used for the U-Net training has no labels and consists only of ground truth samples. As stated before, manually labeling the dataset requires time and effort and was not an option. Instead, an automatic method was desired to be implemented.

## 2. Related Work

Even though most of the researchers use CNNs for image annotation through transfer learning augmented with other techniques [5,6,7,8], several studies regarding this approach can give an overall understanding of the state-of-the-art and the main directions. These studies will be further described in this section, while the conclusions will be compared with our proposed method. In [9], an unsupervised annotation method is proposed for evaluating the snow cover level of roads. The images are collected using 500 open-access traffic cameras installed in Montreal and include different urban areas and snow levels. Four classes are defined as follows: clear, light-covered, medium-to-heavy-covered, and plowed surface. The images undergo a couple of preprocessing steps before being fed to the clustering algorithm. First, the dataset is converted to grayscale. Next, Contrast Limited Adaptive Histogram Equalization (CLAHE) [10] is applied to accentuate the contrast between key details. In the last step, the dataset is binarized so that the details containing snow will be all white and the rest, such as clear roads or cars, will be black. After the preprocessing, a U-shaped convolutional autoencoder (CAE) is used for feature extraction. Its results are flattened and fed into a clustering algorithm based on a detection method called Louvain [11], which outputs clusters that can represent the same defined class. By calculating the cosine matrix of all cluster centers, these are grouped so they will fit the desired classes. In the end, the results are compared against a density-based clustering method, HDBSCAN [12], using four metrics: modularity, coverage, performance, and Dunn’s index. These results outperformed HDBSCAN in all metrics except performance.

The absence of reliable cable object datasets and labels urged for another interesting study that is described in [13]. The study not only generates labels, it also generates input images based on a well-known method from the film industry named chroma-key. First, videos are taken of the target cables over a green background, moving them or changing their places for diversity. Next, using the hue range of the background, the target objects are separated. In the end, various background images can be inserted, augmenting the dataset. Knowing the initial label of the target object, the users do not have to manually annotate every new image or video frame. The main issue of this technique is choosing the background so that it does not color interfere with the target object and alter the training. In this case, green wires and a green initial background are not suited. By applying this method, 28,584 images are generated from 3176 labeled images. Each of the labeled images is randomly overlapped over one of the 15 background images. After that different augmenting techniques are applied such as flipping, shifting, or rotating so that eight new virtual images are generated. In the end, the dataset is tested by feeding it into two deep learning networks: DeeplabV3+ [14] initialized with ResNet 101, and HRNet [15] initialized with ImageNet. Ninety percent of the generated dataset was used for training and the rest for validation. Sixty manually labeled images were used for testing. For accuracy evaluation, the Dice coefficient was calculated. Both the two networks obtained reliable scores of 0.935 and 0.925.

Even though the methods are applied in the medical and microscopic fields, a very recent study from 2023 addressing automated labeling is presented in [16]. The paper attempts to label the Digital Image of Bacterial Species (DIBaS) dataset, which is comprised of 660 images of bacteria. This dataset is frequently used for classification or detection problems in its respective fields, but it has never been used for semantic segmentation before. To obtain the desired goal, a three-step process is implemented. In the first step, the traditional K-means algorithm is performed to obtain centroids for each bacteria species. This was possible due to the staining and uniformity through each species of bacteria. In the second step, Otsu’s thresholding is applied for images containing bacteria similar in color to the background. This method finds an optimal value to obtain a binary image, or to segment the foreground from the background, by minimizing the variance inside the clusters. The third step consists of the morphological closing operation to reduce noise or artifacts such as small gaps within groups of bacteria. The downside of this step was the manual analysis of the closing window, mainly because bacteria species have different sizes. After obtaining the pixel-labeled DIBaS dataset, it was fed into a ResUNet++ network [17], which is a modified U-Net architecture. The accuracy of the F1 score was computed for each of the 33 species. Most of them had around 97% accuracy, with an overall of 95%. In addition, the confusion matrix showed that due to the similarity in dispersion and shape of bacteria, specific species were misclassified and had worse scores.

A widely held notion regarding U-Net is that its effectiveness is contingent upon its distinctive U-shaped architecture, and numerous models inspired by U-Net have been introduced. Regarding the other topic of our study of cost reduction, since its release in 2015, the U-Net model has been constantly modified and trained to enhance accuracy, simplify the architecture, or reduce computational resources. For example, in [18], a low-cost U-Net model called LCU-Net was presented for image segmentation of microorganisms. The paper proposed the same architecture shape as U-Net but augmented with concatenate operations and Inception-V3 [19]. Because the traditional U-Net model is limited to one receptive field of a sigmoid function, the blocks were built using various-size convolutional filters applied on the input and concatenated at the end of each block. Unfortunately, this method increased the number of parameters and each of the convolutional filters of N × N was replaced with two convolutional filters of 1 × N and N × 1. To decompose even further, the Inception approach was utilized by replacing higher-grade convolutional filters with pairs of lower grades. For instance, a 5 × 5 convolutional filter has the same receptive field as two 3 × 3 convolutional filters. Additionally, after each convolutional layer, batch normalization was added, and after each decoder block, a convolutional transpose layer was inserted. The architecture accuracy was tested against the Environmental Microorganism Dataset 5th Version (EMDS-5). The model not only outperformed the original U-Net but also significantly reduced the memory requirement, decreasing it from 355 MB to 103 MB. The evaluation metrics used were Dice, Jaccard, Recall, Accuracy, Precision, and VOE. Except for the last two, all four increased compared with the original U-Net model.

A more recent study of simplifying the U-Net model [20] focused on reducing the complexity of the decoder part. This approach was established by partially deactivating the encoder and decoder blocks and comparing the accuracy with the full structure. The experiment emphasized that training only the encoder obtained comparable results with the full model while using only the decoder for training dropped the performance. The architecture of the intuitively named Half-UNet is composed of five encoder blocks and one decoder block, which aggregates every skip connection through upsampling to the original image size. It is worth noting that the aggregation is done not by concatenation, which requires more computation, but by addition, which helps with the information under each dimension. The model also introduced the ghost module for every block convolution. The ghost model is described in [21] but concisely, it reduces the computation resources by generating half of the feature maps through depthwise separable convolutions. The proposed method was evaluated by computing the Dice coefficient for mammography, lung nodule, endocardium, and epicardium identification. For the endocardium and epicardium, a Half-UNet had better scores and for the first segmentation tasks, the results were similar. Overall, the model reduced the total number of U-Net parameters by almost 150 times.

## 3. Main Contributions

Even though the previously described methods addressed the proposed topics and described useful techniques, they are not well suited for satellite imagery semantic segmentation and have their downfalls such as artificial data generation, user interventions, or supervised algorithms as a starting point. The main contributions of this article can be drawn concerning its two objectives:

- Automatic pixel labeling without any human supervision. This paper proposed a fully unsupervised Gaussian Mixture Model (GMM) technique to label each pixel in one of two classes: forest or no-forest, without any other supervised training, or pre-trained network as a backbone. Compared with other studies, the dataset used is comprised of its own aerial imagery recorded with modern in-house capabilities. This generated a generous real-life dataset which bypasses the need for data augmentation. To prove the success of the GMM technique, the ground truth and its corresponding labels were tested against several U-Net architectures to prove its utility and the utility of the method. The paper showed that the labels were accurate enough so that the U-Net could learn from them and predict good accuracy results without human intervention.
- Reduced U-Net model complexity. Most of the real-life deployments of trained AI models are done in harsh conditions or with very poor hardware capabilities, not to mention the need for fast analyses and predictions. The study conducts tests on simplified U-Net models and obtained similar high-accuracy results while reducing the complexity of the model by 60 times in the best scenario. It is well known that satellite or aerial imagery is composed of vast amounts of pixels due to their field of view (FOV) and high resolution [22]. In conjunction with traditional imagery such as traffic, objects, or writings, semantic labeling takes exponentially more time. Reducing the complexity also reduced the average computation time, resulting in the proposed method labeling and predicting thousands of square kilometers in a reasonable time.

## 4. Dataset and Study Area

Numerous open-source satellite or aerial images can be readily accessed for download on the internet, including platforms like Sentinel or Landsat. However, this research opted to employ a distinct set of aerial images provided by the Geospatial Defense Intelligence Agency (AIGA), an entity under the Ministry of National Defense responsible for supporting troops with geospatial information. This agency utilizes a state-of-the-art airborne digital photogrammetric system known as ADS80, manufactured by Leica Geosystems. The ADS80 system is installed on a specialized cartographic airplane to capture the images. The acquisition process employs a passive push-broom technique, capturing the visual spectrum (RGB) and the near-infrared band (NIR) in four bands, each with an 8-bit radiometric resolution. Despite its inability to record data on other bands such as SWIR [23] or SAR [24], the primary advantage is its high spatial resolution, which may vary depending on the flight altitude but is typically utilized for 50 cm output. Another significant benefit is the clarity of the images, indicating that there are no cloud formations captured in them. This outcome is a result of conducting the flights under clear skies and favorable weather conditions.

After processing the raw data, and considering the institution’s objectives, the images were organized in tiles of 10 km by 10 km. For each tile, two distinct types of products were composed using the four available bands: RGB and CIR (color infrared). RGB images depicted the natural scenery as perceived by the human eye, while CIR images, also called false colors, presented a stack of red, green, and near-infrared bands. The utilization of CIR images proves highly beneficial, as they offer precise and valuable information about the natural environment. For example, brighter infrared regions signify denser and healthier vegetation, while man-made objects are depicted using tones of blue or green.

**Figure 1:**
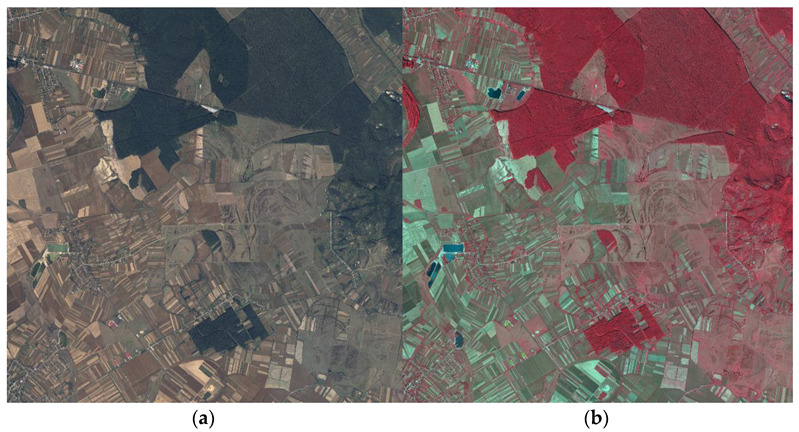
Samples of the aerial imagery. (**a**) RGB image; (**b**) CIR image.

Taking into consideration the segmentation purpose of the study, AIGA provided four tiles of 100 km^2^ each recorded near Targu-Mures city of Romania, an area abundant in forests, but also mixed with other land cover classes such as settlements, crops, grazing lands, or roads. Two samples of the provided data are shown in Figure 1.

Given that both RGB and CIR images share common red and green bands, a preprocessing step is essential to eliminate repetitive data while preserving relevant information. Initially, the RGB images were split into three distinct bands. Then, from the CIR images, only the first band, which represents near-infrared, was extracted. Subsequently, all four extracted bands were combined and saved as separate array files, resulting in the creation of new RGBN images. Additionally, the file format was converted from TIFF to NumPy extension to facilitate further analysis and manipulation.

In the end, four 20.000 × 20.000 × 4 arrays were created. The previous work presented in [25] was applied to construct the final dataset. In the previous study, various scenarios were tested and it was found that the best input dimensions for a two-class GMM clustering were 4000 × 4000 × 4. The four arrays were cut into 25 smaller areas, resulting in a 100 4-band image dataset.

## 5. Methodology

### 5.1. Overall Algorithm Diagram

**Figure 2:**
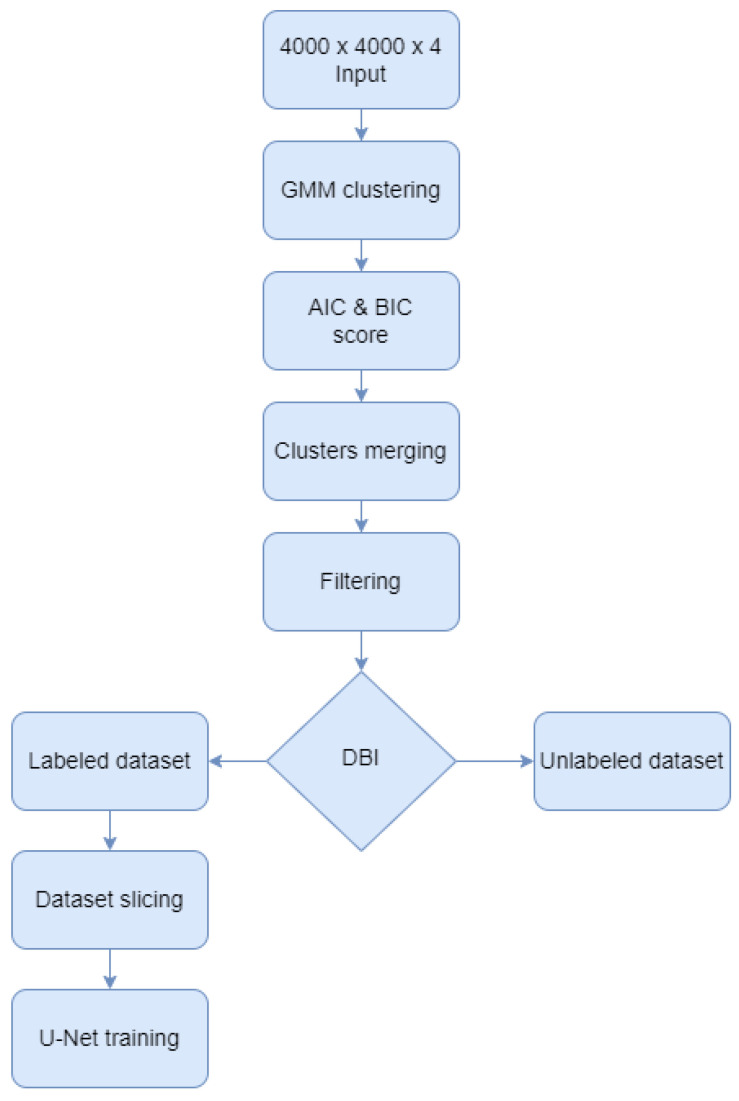
Algorithm diagram.

This paper proposed a Gaussian Mixture Model [26] approach for semantic labeling of the 100 aerial images dataset, before training U-Net architectures for forest classification. As shown in Figure 2, the GMM algorithm was augmented with several techniques to enhance the computed labels such as calculating information criteria, merging classes, and filtering the output images. After the GMM was computed, the Akaike information criterion (AIC) and the Bayesian information criterion (BIC) scores were calculated to validate the optimal number of clusters. Next, the resulting clusters were merged to form only the two classes of the study: forest and no-forest. After filtering the results, the Davies-Bouldin index was used to select the most reliable images and their labels to be the foundation of the U-Net training, validation, and testing dataset. The rest of the unchosen GMM results remained unlabeled data which can be used for prediction and visual interpretation in the future. In the end, the labeled dataset was sliced and fed into 19 convolutional layers and 4 skip connections architectures. Each of these steps is further detailed.

### 5.2. GMM Clustering

The starting point of this step was the previous work where meaningful parameter tuning was performed. Regarding the number of bands, the study proved that the near-infra-red band improved the unsupervised clustering. It is worth noting this is not an obvious remark because in aerial imagery there was plenty of healthy vegetation, not just forest, and this can interfere with the clustering. Several tests were conducted concerning the input dimension for the GMM algorithm. The input dimension is important because this determines how closely or distantly the algorithm “perceives” the ground truth that you intend to present. For a two-class clustering, the best results with an accuracy of 92.22% were the 4000 × 4000 4 band inputs.

**Figure 3:**
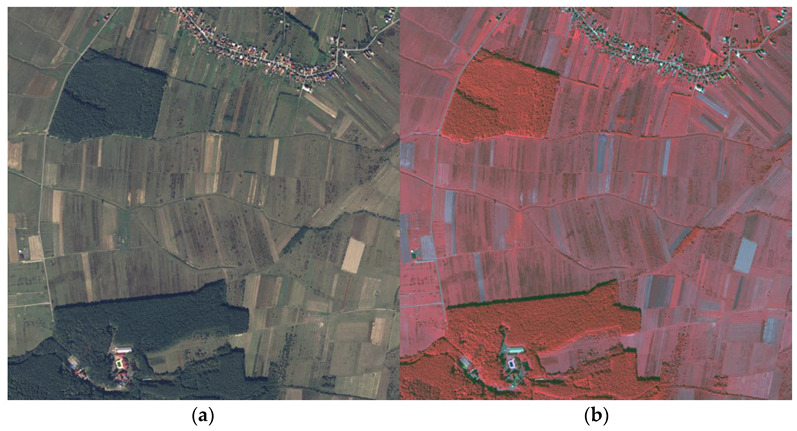
GMM training image. (**a**) RGB image; (**b**) CIR image.

Taking all of the above into consideration, the present study proposes a more extensive implementation that addresses one remaining issue. Even though the final purpose is to identify two classes, and the forest indeed is dominant in the dataset, finding more clusters within the data was implemented. The image chosen for training and finding the centers of the clusters is depicted in Figure 3a. It contained major land covers such as forests, orchards, settlements, crops, bare lands, irrigation channels, or roads. On the right, it was observed that the forests have the highest NIR intensity which will be an important factor during clustering.

**Figure 4:**
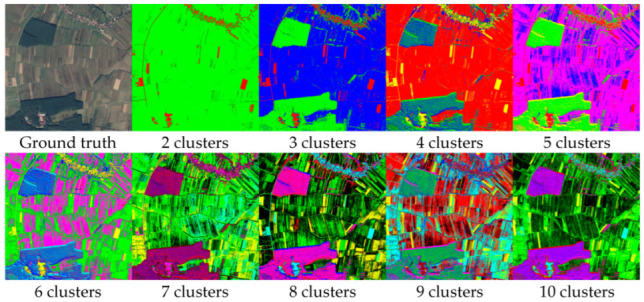
Results of GMM-based clustering.

The GMM algorithm was modified to search for two to ten clusters. In Figure 4, the clustering results are shown for each number of components, the first one being the ground truth. The colors representing each cluster were chosen randomly and eccentrically for a better visual experience. While the two-cluster segmentation was unable to properly identify the forest, the rest of the iterations had decent results. Unfortunately, starting with the 6th iteration, the results become granulated and the target classes were too mixed. In some areas, the forest was even represented in four distinct colors, which is far from reality.

To further analyze the best scenario, the AIC [27] and BIC [28] scores were implemented. These two are widely employed criteria for model selection in clustering, aiding in identifying the optimal number of clusters within a dataset. Grounded in information theory principles, these criteria strike a balance between model fit and model complexity, facilitating the determination of the most suitable clustering solution. This solution is also proposed in [29] where the appropriate GMM number of clusters was decided to identify the driving style of car drivers.

The primary distinction between AIC and BIC lies in their treatment of the trade-off between goodness-of-fit and complexity. AIC adheres to the maximum likelihood principle and penalizes models with a substantial number of parameters relative to the data size. On the other hand, BIC, similar to AIC, takes into account the number of parameters but imposes a more rigorous penalty on models with a larger number of parameters. Let L be the maximum likelihood function, k be the number of the parameters in the model, and N be the number of points. Then AIC and BIC had the following formulas:

(1) AIC=2k−2ln⁡(L)

(2) BIC=kln⁡(N)−2ln⁡(L)

The maximum likelihood [30] of a model corresponds to the set of parameters that maximizes the likelihood function which is calculated as a function that quantifies the probability of the observed data given the model’s parameters. In our case of GMM clustering, the Gaussian likelihood function was implemented in (3), where $y_{i}$ are the real targets and ${\hat{y}}_{i}$ are the predicted targets, but there are other functions such as Poisson or Bernoulli.

(3) $$\log\left(L\right)=-\frac{N}{2}\log\left(2\pi \right)-\frac{N}{2}\ln{(\sigma }^{2})-\frac{\sum_{i=1}^{N}{\left(y_{i}-{\hat{y}}_{i}\right)}^{2}}{{2\sigma }^{2}}$$

**Figure 5:**
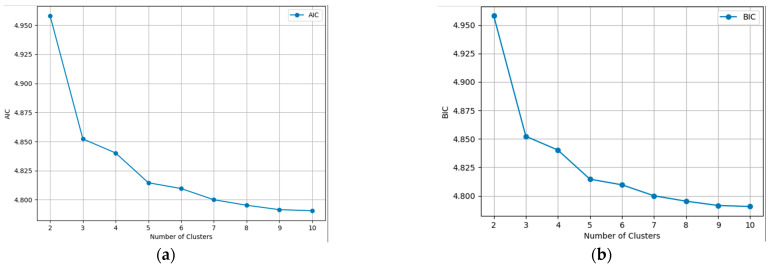
Information criterion values for each number of clusters. (**a**) AIC; (**b**) BIC.

In Figure 5 the evolution for every clustering scenario is presented, dealing with such large image results in large calculated values. Even though the two charts might look identical, the Y axis was divided by 10^8^ for the virtue of plotting. In theory, the best scenario is the one with the lowest AIC or BIC score, but in semantic aerial imagery, this can lead to never-ending processes and overfitting. To obtain the balance between performance and accuracy, we applied the principle of the Elbow method, which states that the optimal number of clusters is the point where the within-cluster sum of squares decreases more slowly, or, in our case, the AIC and BIC scores. It is obvious from the graphics that from six to ten, the differences were smaller, so the question was whether four or five clusters were the better approach. Comparing the two results from Figure 5, it can seen that the algorithm identified a fifth distinct cluster. The agricultural land was clustered in three parts, instead of two, depending on its texture and cover, and the forest was segmented into the same two clusters. Taking all of the above into consideration, it was established that four is the optimal number of clusters.

### 5.3. Clusters Augmentation

**Figure 6:**
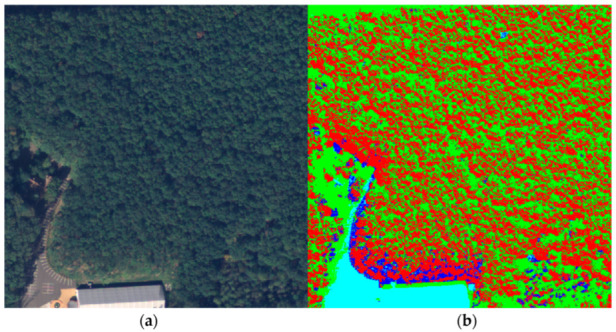
Forest sample. (**a**) Ground truth; (**b**) Clusters.

For the next step of the workflow, we predicted the four clusters for the whole 100-image dataset using the centers calculated by the GMM. Also, these outputs were collapsed into two classes, one that represented the forest and the other that represented any other entities present in the image. For this, the forest texture must be observed closely. Due to the different heights of the trees in the forest, shadows were generated, and a large portion of the pixels were darker and were grouped in a different cluster, making the forest pixel values not uniform. Figure 6 emphasizes this issue by illustrating a forest sample (a) and its corresponding clusters (b) from the training image.

**Figure 7:**
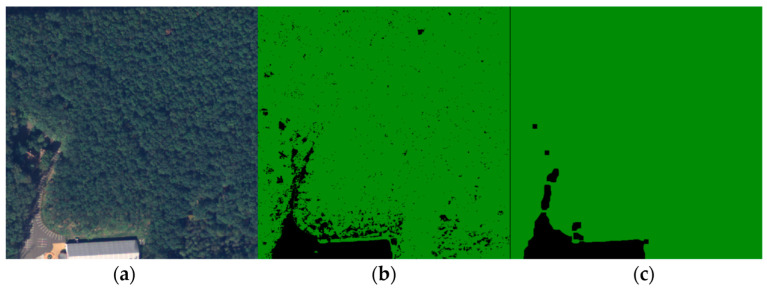
Image filtering. (**a**) Ground truth; (**b**) Merged clustered image; (**c**) Filtered image.

These two clusters describing forests were merged, while the other two describing crops, bare lands, or settlements were also collapsed into one class. Another important feature of the forest’s texture is the mixture of deciduous and conifer tree species. Working with images from a high-altitude area, the conifers, which have a brighter NIR footprint, were predominant. On the other hand, deciduous or withered trees found inside large forest areas, having a lower NIR footprint, were clustered as barren land or grassland. This created small voids inside forest areas as shown in Figure 7b. To address this issue properly, two filtering steps were applied to the merged images: a median filter and a closing filter, both utilizing a 9-pixel kernel. The median filter was mainly applied to reduce overall noise and various artifacts present in the image. The closing filter was constructed from two operations [31]. The first was dilation, which produced the maximum value if at least one pixel within the kernel possessed this value. In our case, this was the forest because the color values used for representing the two classes were (34, 139, 34) and (0, 0, 0). Consequently, dilation expanded the forest area. On the other hand, the erosion operation worked in the opposite direction. It eroded the boundaries of the foreground object, which aided in removing the mentioned holes.

Considering the fundamental need for precise labels for the supervised training, the next step of the algorithm was analyzing the clusters’ dispersions from two points of view, compactness, and separation. While compact clusters are desired, they should also be well-separated from each other. The mathematical tool suitable for this is the Davies–Bouldin index which takes into account both the intra-cluster dispersion (how compact the data points are within a cluster) and the inter-cluster distance (how far apart clusters are from each other). The index has the following formula:

(4) $$DB=-\frac{1}{N}\sum_{I=1}^{N}{max}_{j\neq i}(\frac{\sigma_{i}+\sigma_{j}}{d\left(c_{i},c_{j}\right)})$$

where $c_{i}$ is the center of cluster *i* and $d\left(c_{i},c_{j}\right)$ is the distance between the centers of clusters *i* and *j*. After calculating the index for all 100 images, the best 16 were selected to become labels of forest and no-forest areas. The lower the index, the better the clustering result.

**Figure 8:**
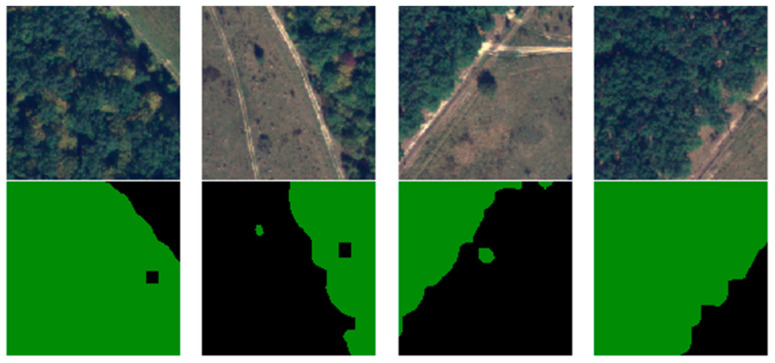
Samples of labeled training dataset.

Due to the 4000 × 4000 × 4 dimensions of the images, these could not be fed entirely into the architecture. The dimensionality was also a problem regarding the labels because semantic segmentation labeled every pixel instead of an entire image. Taking into consideration that the U-Net commonly requires square powers of two to be fed, the 16 images were cut into 128 × 128. For practicality, the pixels from 3841 to 4000 were discarded, resulting in 900 samples per image and a total of 14,400 samples composed of the ground truth and their labels. The other 84 unselected images were also split, but without the two-class segmentation, and treated as unlabeled data. From the 14,400 samples, 10.000 were used for training and validation, and 4400 for testing. Conventionally, 10% of the dataset was used for testing, but the desire was to obtain correct, meaningful results, and have a better understanding of the model generalization. In object classifications, having a too-small dataset might pose some problems, but for semantic classifications, every pixel is a learnable feature for the model. Figure 8 illustrates several training samples and their labels.

## 6. The Original U-Net Architecture

Since its release in 2015, the U-Net architecture has gained widespread popularity as a CNN mainly employed for image semantic segmentation purposes. We chose this architecture over others for several reasons. The U-Net employs an Encoder-Decoder architecture, comprising two main paths. The encoder path systematically reduces the spatial dimensions of the input image, thereby extracting higher-level features. On the other hand, the decoder path restores the encoded features by upsampling, leading to an enhanced segmentation mask with refined spatial resolution. This unique architecture enables the network to grasp both the broader context information and intricate local details during the learning process.

**Figure 9:**
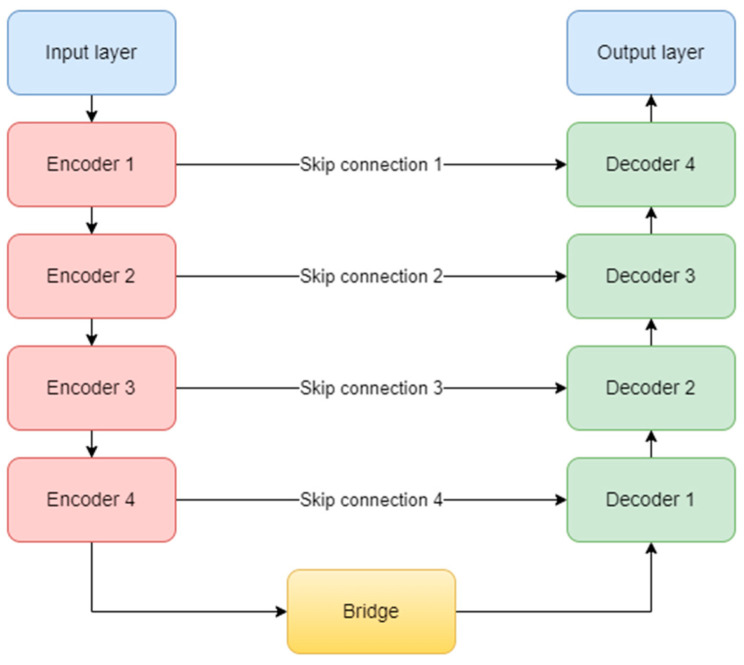
U-Net architecture.

The original complex architecture is composed of one input layer, four encoder blocks, one bridge block, four decoder blocks, four skip connections, and one output layer (Figure 9). It uses the ReLU (Rectified Linear Unit) activation function for all the layers in the model, except the last one, which obviously, needed a sigmoid function [32]. At the end of the encoding path, a dropout layer is used to prevent overfitting.

Each encoder block consists of two convolutional layers and one max-pooling layer. The convolutional layer uses a 3 × 3 kernel and is not padded, but in our study, we implemented zero padding. In this way, the input dimensions are kept the same throughout the model. The number of feature maps doubles, starting with 64 for the first block, and up to 512 for the fourth block. The max-pooling layer is applied at the end of each encoder block to reduce the number of parameters and computation time and to retain only the most prominent information of the feature maps. Moving forward, the linkage to the decoder is done through the bridge block composed only of two convolutional layers of 1024 feature maps.

**Figure 10:**
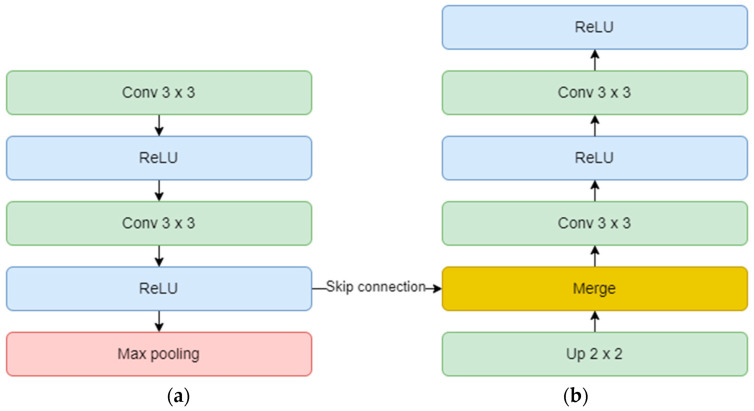
Encoder (**a**) and decoder (**b**) block diagram.

On the other hand, the decoder aims to recover the spatial resolution of the input by having one upsampling layer at the start of each block. This layer has a size of 2 × 2, translating to doubling the width and height of the previous layer, and it also uses zero padding, instead of no padding. Next is the concatenation layer which implements the skip connection technique. The incorporation of skip connections facilitates the decoder in utilizing feature maps from earlier encoder layers, ensuring the preservation of spatial information and mitigating the vanishing gradient problem. These connections play a critical role in achieving accurate segmentation by allowing the network to combine low-level and high-level features, thereby aiding in precise boundary delineation. The concatenation layer merges one convolution from the encoder block with the previous upsampling. In the end, two convolutional layers with the same number of feature maps, but backward are added. The overall diagram of an encoder (a) and a decoder (b) block is depicted in Figure 10. Finally, the output layer operates on the features obtained from the decoder. In essence, it functions as a convolutional layer with a single feature map and a 1 × 1 kernel. It condenses these features into a single-channel binary segmentation mask, which can be interpreted as a probabilistic map. By applying the sigmoid activation function, the pixel intensities are transformed into probabilities. The ultimate output is a segmentation mask that represents the likelihood of each pixel belonging to either of the classes.

## 7. Non-Structural Hyperparameters Tuning

The multitude of hyperparameters of a CNN can be divided into two groups. The structural hyperparameters are the ones presented during the previous architecture description: number of convolutional layers, number of kernels, kernel size in each convolution, activation functions, and pooling sizes. On the other hand, the optimizer, the learning rate, the batch size, the number of epochs, and the loss function, constitute the non-structural hyperparameters and need specific tuning for our purpose.

In the case of U-Net used for segmentation tasks, the loss function evaluates how well the predicted segmentation mask aligns with the true segmentation mask for a set of input images. It measures the dissimilarity between the probability distribution of the predicted pixel values and the corresponding actual binary labels. There are various choices, but the best fit for the scope of the study is binary cross-entropy. This is calculated for each pixel using the following formula:

(5) $$LL=-\frac{1}{N}\sum_{i=1}^{N}y_{i}(\log\left(p\left(y_{i}\right)\right)+(1-y_{i})\log\left(1-p\left(y_{i}\right)\right)$$

where $y_{i}$ is the true binary label and $p\left(y_{i}\right)$ is the predicted probability of the sigmoid.

Optimizers hold significant importance within the training process as they steer the parameter updates. Their role involves dictating the magnitude of parameter adjustments based on computed gradients of the loss function concerning these parameters. These gradients serve as indicators of the optimal direction in which the parameters should be modified to minimize the error. In essence, optimizers orchestrate the intricate dance of parameter fine-tuning by intelligently scaling the steps taken toward the optimal solution. They ensure that the model converges toward a more accurate representation of the desired output, aligning its predictions with the actual target values as the training progresses.

Fortunately, in [33], extensive work was presented to classify the best optimizers for brain tumor segmentation. The best results were obtained using Adaptive Moment Estimation (ADAM) [34], which we also implemented over the Stochastic Gradient Descent (SGD) found in the original U-Net study. ADAM optimization integrates the advantages of two other optimization techniques, Momentum, and RMSProp, yielding an enhanced approach. The Momentum algorithm employs past gradients to alleviate optimization fluctuations, whereas RMSProp adjusts the learning rate based on recent gradient magnitudes. ADAM optimization elevates these concepts by introducing an exponential moving average of both gradients and their squares, dynamically fine-tuning the learning rates. In practical terms, ADAM optimization computes ongoing averages of gradients and squared gradients for each model parameter. These averages are harnessed to compute parameter updates during training. In a nutshell, ADAM works as follows:

1. Calculate the gradient ∇θJ(θ);
2. Update biased first moment estimate:
  (6) $$m_{t+1}=\beta_{1}m_{t}+(1-\beta_{1})\nabla \theta J(\theta )$$

3. Update biased second moment estimate:
  (7) $$v_{t+1}=\beta_{2}v_{t}+(1-\beta_{2})({\nabla \theta J(\theta ))}^{2}$$

4. Correct bias in the first and second moment:
  (8) $${\hat{m}}_{t+1}=\frac{m_{t}}{1-\beta_{1}^{t}}$$
  (9) $${\hat{v}}_{t+1}=\frac{v_{t}}{1-\beta_{2}^{t}}$$

5. Update the parameters:

(10) $$\theta =\theta -\propto \frac{{\hat{m}}_{t+1}}{\sqrt{{\hat{v}}_{t+1}+\epsilon }}$$

where J(θ) is the loss function, ∝ is the learning rate, $\beta_{1}=0.9$ and $\beta_{2}=0.999$ decay rates kept constant in our study, and ϵ a very small number to prevent division by zero.

In tuning the rest of the hyperparameters, one must consider the computation power. The machine used was a 7th generation i7 with a 3.8 GHz maximum frequency, 32 Gb DDR4 memory, and 4 Gb GDDR5 nVidia GeForce GTX 1050 Ti laptop. Unfortunately, these are not state-of-the-art resources. To tackle the issue, the batch size, which determines the number of samples processed in one forward and backward pass during each training iteration, was set to the maximum possible. This was achieved by constantly increasing it until the system returns an OOM (Out of Memory) error. For this analysis, we also set the model to compute only one epoch and monitored the computation time. The learning rate used was 10^−5^ but it was not relevant to this step analysis. The results are shown in Table 1. They are comparable because an identical seed was used to randomize the dataset. The largest batch size accepted by the GPU was 16, which also had the best computation time.

**Figure 11:**
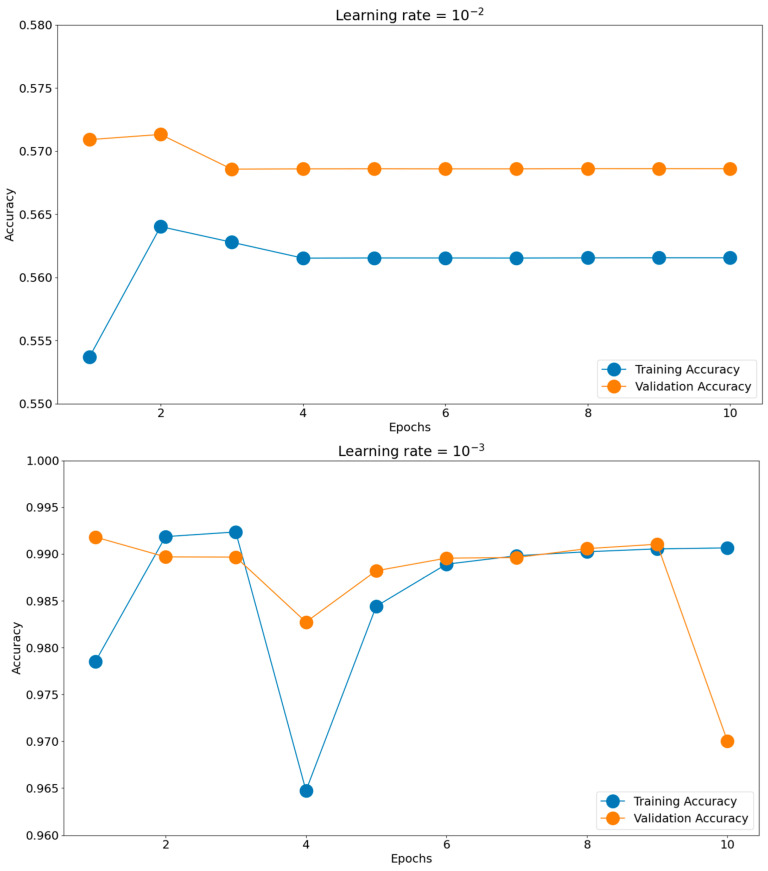

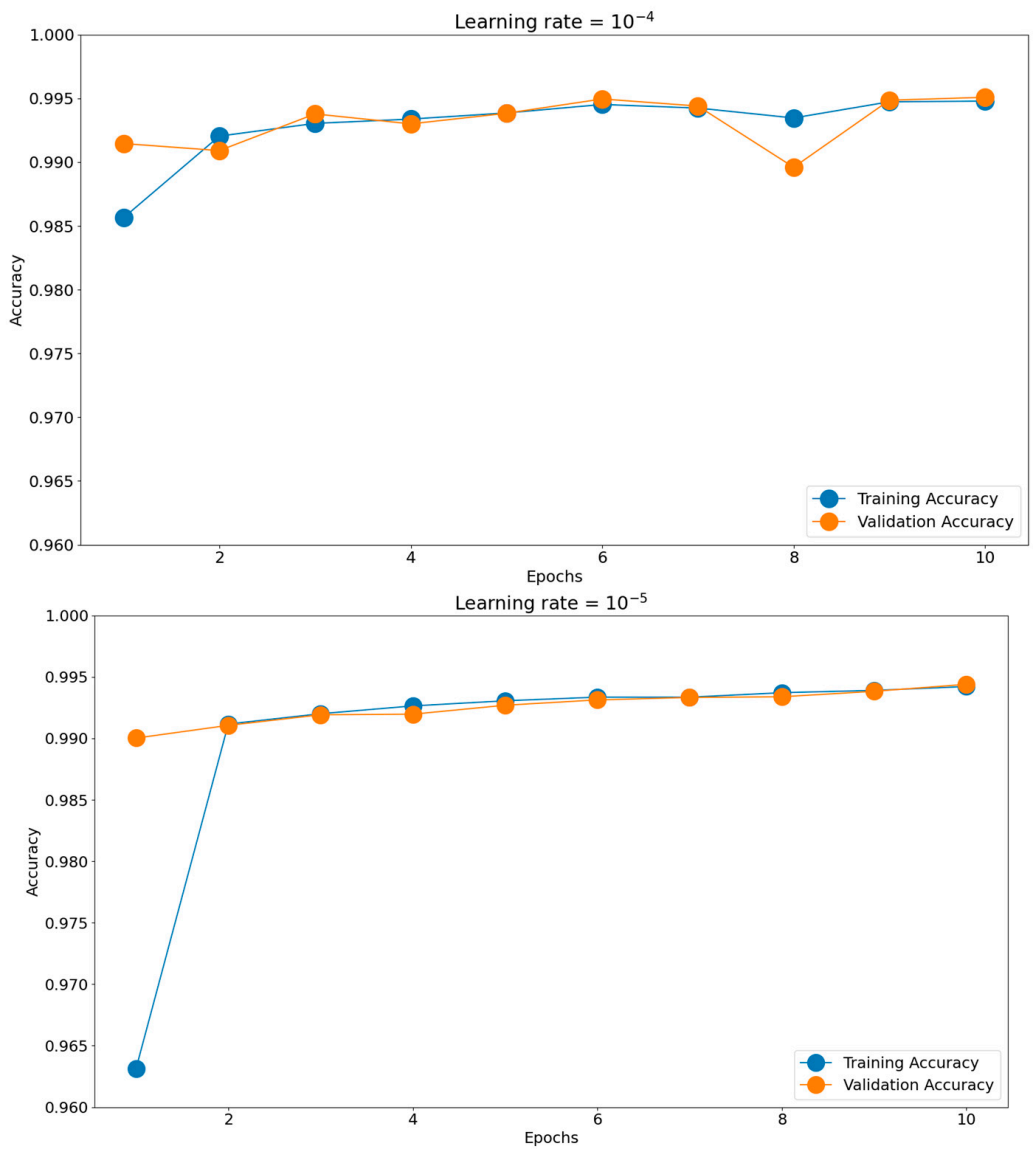
Training histories for different learning rates.

Having such good initial accuracies, we concluded the rest of the tuning to be done on 10 epochs of micro training and only the best-found scenario to be iterated, if necessary, for more epochs. Establishing the batch size and optimizer took us toward the next hyperparameter, the learning rate. The chosen learning rate holds the potential to impact the generalization proficiency of the trained model when applied to unfamiliar data. Should the learning rate be excessively elevated, the model might excessively conform to the training data, impeding its ability to generalize effectively when confronted with novel data. There is a consensus to train models using these negative powers of 10. Opting for an appropriate learning rate enables a harmonious equilibrium to be reached, enabling the model to appropriately fit the training data while mitigating the risk of overfitting. This can be observed in Figure 11, where the training and validation accuracies for the most common four learning rates are presented: 10^−2^, 10^−3^, 10^−4^, and 10^−5^. The best score was achieved for a learning rate of 10^−4^, which obtained the highest validation accuracy of 0.9951. The validation accuracy was also maintained closer to the training accuracy through all 10 epochs. The 10^−2^ learning rate badly underperformed, obtaining accuracies around 0.56, while the 10^−3^ learning rate had more scattered validation accuracy values compared to the training graph. In the end, the 10^−5^ learning rate was slower in converging than 10^−4^, having lower accuracy scores for both the training and validation datasets.

## 8. Results

### 8.1. U-Net Model Downscaling

Having all the non-structural hyperparameters set for the original U-Net model, we established the starting point of the architecture downscaling, by subsequently reducing the number of kernels present in each convolution layer by half. Several were created while maintaining the doubling and symmetrical rules of the U-Net model. The complexity of each block (number of kernels per convolution layer) and the trainable number of parameters are described in Table 2.

Following the training of each scenario with the predefined hyperparameters, the evaluation process involved two key aspects: computation time and the accuracy of predictions on the testing dataset. The accuracy of predictions was compared against the performance of the original U-Net model (first scenario) to gauge the extent of any significant deviations. The outcomes of these assessments are outlined in Table 3. The “total time” metric signifies the duration required to predict all 4400 images, while the “average time” metric exposes the computation time for a single image. The last column emphasizes the decrease in time from the initial scenario. The original scenario obtained a 0.99688 accuracy and it took, on average, 0.14 s to successfully make a prediction. Going further, the next three downscaling scenarios of the model had similar accuracies with a much faster computation time. A meaningful decrease in accuracy was spotted in the fifth scenario, which had 16 times fewer feature maps on every convolution layer than in the first scenario.

Because the testing dataset had 4400 samples, the overall calculated differences could have been reduced or could falsely emphasize discrepancies. To further analyze the simplified models’ performances, we also monitored the memory usage, accuracy, and computation time for a single random image and illustrated them in Table 4. Compared with the original model’s fairly large memory usage of 650 Mb, the next three downscaling scenarios performed at a similar accuracy, but with lower resource consumption. The last two scenarios also had good results, but the accuracy continued to decrease without a significant memory improvement. It can also be seen that scenarios 4 to 6 had a memory usage decrease of around 95% while maintaining high accuracy.

**Figure 12:**
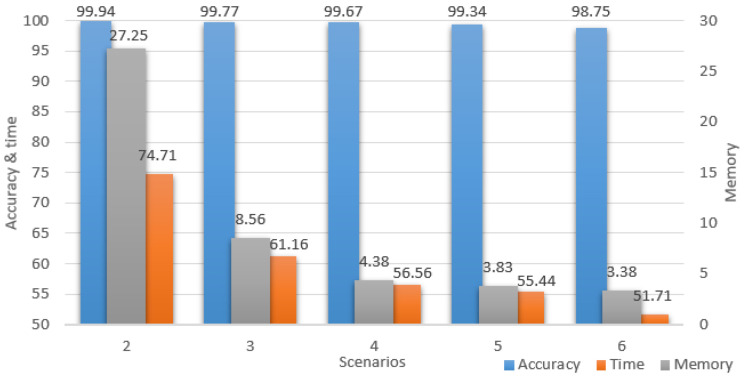
Scenarios performances.

For a better understanding of the study improvements, all the performance results for all the downsized scenarios are portrayed in Figure 12, where all the values represent percentages of the initial scenario. The chart has two vertical axes, the accuracy and time are projected on the left, while the memory is projected on the right. It is important to notice the accuracy maintained high percentages while the time and memory percentages constantly decreased up to the point where the model computed twice as fast and used only 3.38% of the initial memory.

### 8.2. Comparison with Related Work

Even though there were different datasets and segmentation purposes, a couple of comparisons can be made with the related work presented at the beginning of the paper. The comparison was done in two directions regarding the purposes of this study. The work described in [13,16] can be correlated with obtaining high-accuracy classification models without manual data labeling. On the other hand, the work described in [18,20] focused on reducing U-Net memory needs and parameters, while maintaining its accuracy.

In Table 5, the F1 score is presented for each of the studies. The chroma-key method was evaluated using two deep learning networks, DeepLabV3+ and HRNet, while the semi-automatic DIBaS dataset labeling was tested through ResUNet++, which is a U-Net model using deep residual learning. For our study, we computed the F1 score for the fourth and sixth scenarios, because the fourth was the most balanced and the sixth had the lowest accuracy. Our proposed method had better results by a margin. We suspect this was a consequence of an abundant dataset and an enhanced clustering phase. Unfortunately, ResUNet++ had low results regarding the F1 score, even though it had an overall accuracy of 0.95. Because the study segmented more than 30 classes of bacteria, some of the species had a high grade of similarity or arrangement, increasing the false positives and decreasing the F1 score. Our study bypassed this issue of multi-class segmentation by aggregating similar classes to isolate the one of interest.

A more precise comparison can be assessed regarding the complexity of the proposed method. The memory usage and total number of parameters and filters of LCU-Net, Half-UNet, and our scenarios are presented in Table 6. Even though LCU-Net had the same number of blocks and filtering increments (16 to 256 and back) as the third scenario, due to the implementation of the Inception approach, the total number of filters and parameters was much higher. On the other hand, Half-UNet had 6 blocks with 64 kernels each because it deactivated 3 of the 4 decoder blocks, resulting in only 212.576 parameters. Except for the second scenario, our proposed method had better cost-reduction results. The third scenario managed to use only 55.7 Mb compared with 103.5 Mb of LCU-Net and 137.3 Mb of Half-UNet. Regarding the average time, this measure can be highly affected by the hardware resources. No data was presented for Half-UNet but LCU-Net predicted the results on average in 0.15 s using an Intel(R) Core(TM) i7-8700 CPU with 3.20 GHz, 32 Gb RAM, and Nvidia GeForce RTX 2080 8Gb. Our workstation had a similar CPU and memory, but a 4 Gb video card, and due to the lower number of parameters, obtained results below 0.10s.

## 9. Conclusions

This work addressed a common issue of neural network training, the lack of labeled data, and the downfalls of obtaining it. To tackle the problem, this paper studied the use of the Gaussian Mixture Model technique for automatic data labeling and implemented a workflow for obtaining consistent data for supervised training. The method was applied for a current issue, deforestation monitoring, by semantical segmenting forests on aerial imagery. The dataset was used to train a conventional 9-block U-Net model for algorithm assessment. Benefiting from the dataset’s quality, the resulting training, validation, and testing accuracies were notably high. The non-structural hyperparameters such as the learning rate, the optimizer, or the batch size had uncommon flexibility, proving the prior GMM clustering enhanced the supervised training even more. The model achieved a validation accuracy of 0.9951, and a testing accuracy of 0.9969 only by micro-training for 10 epochs.

Additionally, the study addressed the problem of resources and computation time of CNN model predictions. A trained architecture needs to output precise results and consume as few resources as possible to be suitable in a real-life environment. The original U-Net model has 31.032.321 parameters and 6848 kernels. Even though it obtains a high accuracy, other satisfactory accuracies can be reached with less complexity. For this, six simplified U-Net models were trained and used for prediction. This study showed that the initial accuracy had insignificant decreases while consuming much less memory. The most balanced scenario was the fourth, which had an 8-kernel first encoder block and a 32-kernel bridge. Compared with the original model, the scenario not only managed to predict the testing dataset two times faster (56.56%), with almost no overall accuracy decrease, but it used only 4.38% of the initial needed memory. The last two scenarios also showed that the U-Net model can be simplified even further for harsh computational power if the accuracy decrease tolerance is higher. Even though the decrease in accuracy was not high, the improvement in time (≈5%) or memory (≈1%) from the fourth scenario was not meaningful. Compared with related work, our proposed scenarios outperformed them in both accuracy and cost reduction categories. The F1 score comparison is fairly volatile, but the GMM clustering on abundant data proves the point of enhanced accuracy. Concerning the complexity, we can assume that reducing the number of filters while keeping the original U-Net architecture can potentially have better results than implementing other techniques such as ghosting or inception to diminish the number of parameters.

In conclusion, we can strongly affirm that the GMM method helps in data labeling and augmentation, and computation resource reduction for supervised training, and future analyses using smaller datasets or lower-resolution sensors should be made to further research its utility. Regarding the hyperparameters optimization, this study showed that an 856-kernel U-Net model has a desirable accuracy and decreases memory usage by 95.62%, obtaining in the process a low-cost model for semantic segmentation.

## Figures and Tables

**Table 1:** Computation time for multiple batch sizes.

Batch Size	2	4	8	16
Time (s)	1502	1112	805	520

**Table 2:** Downscaling scenarios.

Scenario No.	1 of 6	2 of 6	3 of 6	4 of 6	5 of 6	6 of 6
Encoder 1	64	32	16	8	4	2
Encoder 2	128	64	32	16	8	4
Encoder 3	256	128	64	32	16	8
Encoder 4	512	256	128	64	32	16
Bridge	1024	512	256	128	64	32
Decoder 1	512	256	128	64	32	16
Decoder 2	256	128	64	32	16	8
Decoder 3	128	64	32	16	8	4
Decoder 4	64	32	16	8	4	2
**Total kernels**	6848	3424	1712	856	428	214
**Trainable parameters**	31,032,321	7,760,385	1,941,249	485,889	121,761	30,585

**Table 3:** Overall downscaling evaluation.

Scenario	Testing Acc.	Total Time (s)	Average Time (s)	Decrease (%)
1	0.99688	626.51	0.14239	-
2	0.99669	468.05	0.10637	25.29
3	0.99678	383.17	0.08708	38.84
4	0.99601	354.36	0.08054	43.44
5	0.99141	347.35	0.07894	44.56
6	0.99139	323.95	0.07362	48.29

**Table 4:** One sample downscaling evaluation.

Scenario	Time (s)	Acc.	Mem. Usage (Mb)	Decrease (%)
1	0.09373	0.99610	650.7	-
2	0.06247	0.99548	177.3	72.75
3	0.06246	0.99377	55.7	91.44
4	0.06249	0.99280	28.5	95.62
5	0.04686	0.98950	24.9	96.17
6	0.04684	0.98364	22	96.62

**Table 5:** F1 score comparison.

Model	DeepLabV3+	HRNet	ResUNet++	Scenario 4	Scenario 6
F1 score	0.935	0.925	0.77	0.996	0.991

**Table 6:** Complexity comparison.

Model	Filters	Mem. Usage (Mb)	Average Time (s)	Parameters
LCU-Net [18]	5392	103.5	0.15	3469.393
Half-UNet [20]	768	137.3	-	212.576
1 (U-Net)	6848	650.7	0.142	31,032.321
2	3424	177.3	0.106	7760.385
3	1712	55.7	0.087	1941.249
4	856	28.5	0.081	485.889
5	428	24.9	0.079	121.761
6	214	22	0.074	30.585

## Data Availability

The data used in this paper is the property of the Geospatial Defense Intelligence Agency. The dataset was provided for scientific research purposes only under a written agreement. The aerial imagery is not open-source but can be acquired for a specific price.
